# Identification of skin-expressed genes possibly associated with wool growth regulation of Aohan fine wool sheep

**DOI:** 10.1186/s12863-014-0144-1

**Published:** 2014-12-16

**Authors:** Nan Liu, Hegang Li, Kaidong Liu, Juanjuan Yu, Ran Bu, Ming Cheng, Wei De, Jifeng Liu, Guangling He, Jinshan Zhao

**Affiliations:** Qingdao Agricultural University, Qingdao, 266109 China; Qingdao Institute of Animal Science and Veterinary Medicine, Qingdao, 266100 China; China Agricultural University, Beijing, 100193 China; Nanjing Medical University, Nanjing, 210002 China; State key Laboratory of Hydroscience and Engineering, Beijing, 100084 China

**Keywords:** Microarray, Proteomic technology, Wool growth, Differential expression

## Abstract

**Background:**

Sheep are valuable resources for the animal fibre industry. Therefore, identifying genes which regulate wool growth would offer strategies for improving the quality of fine wool. In this study, we employed Agilent sheep gene expression microarray and proteomic technology to compare the gene expression patterns of the body side (hair-rich) and groin (hairless) skins of Aohan fine wool sheep (a Chinese indigenous breed).

**Results:**

Comparing the body side to the groin skins (S/G) of Aohan fine wool sheep, the microarray study revealed that 1494 probes were differentially expressed, including 602 more highly expressed and 892 less highly expressed probes. The microarray results were verified by means of quantitative PCR. Cluster analysis could distinguish the body side skin and the groin skin. Based on the Database for Annotation, Visualization and Integrated Discovery (DAVID), 38 of the differentially expressed genes were classified into four categories, namely regulation of receptor binding, multicellular organismal process, protein binding and macromolecular complex. Proteomic study revealed that 187 protein spots showed significant (p < 0.05) differences in their respective expression levels. Among them, 46 protein entries were further identified by MALDI-TOF/MS analyses.

**Conclusions:**

Microarray analysis revealed thousands of differentially expressed genes, many of which were possibly associated with wool growth. Several potential gene families might participate in hair growth regulation. Proteomic analysis also indentified hundreds of differentially expressed proteins.

**Electronic supplementary material:**

The online version of this article (doi:10.1186/s12863-014-0144-1) contains supplementary material, which is available to authorized users.

## Background

Sheep are valuable resources for the animal fibre industry. Identifying genes which regulate wool growth offers the opportunity to improve the wool production efficiency, product quality and diversity in breeding programs. It can also offer the opportunity to develop transgenic lines and to develop therapeutic agents that can be used to tailor for desirable fibre attributes by altering gene expression [1]. The genetic polymorphisms and their mechanisms of wool and cashmere growth and regulation have been thoroughly studied [1-5]. In mammals, several gene families, such as *WNTs, tumor necrosis factors (TNFs), fibroblast growth factors (FGFs) and transforming growth factor(TGFs)*, have been implicated in hair follicle initiation, morphogenesis and cycling [6,7]. Recently, the molecular characteristics of primary wool follicle initiation in Merino sheep have been reported recently [8].

Transcriptomic research such as microarray analysis, has been successfully applied to investigate the characteristics of hair follicle stem cells in mice [9-12]. Microarray studies have been reported for different traits in sheep and goats, such as resistance to parasites [13,14], mammary development and milk quality [15-17], wool follicle development [18], natural fleece rot resistance [19] and pigmentation traits of skin and wool [20]. A subset of skin-expressed microRNAs with possible roles in goat and sheep hair growth has also been reported [21]. Several studies have demonstrated the usefulness of cDNA microarray for expression profiling of wool follicle growth cycles in whole skin [22-24]. More recently the RNA-seq method was also used to determine the genes differentially expressed among various tissues (including whole skin) of sheep [25].

The Aohan fine wool sheep, bred in Inner Mongolia, is an outstanding breed, providing both wool and meat. The major characteristics of this breed are high quality wool,high disease resistance, and high adaptability. According to figures, Aohan fine wool sheep can provide up to 9 kg of quality wool per year (fiber length up to 10.5 cm, fibre diameter less than 22 μm). Therefore, Aohan fine wool sheep are considered as a valuable genetic resource for fine wool production. Wang et al. demonstrated that seasonal factors significantly influenced the wool growth of Aohan fine wool sheep [26]. The peak of the growth rate occurs in summer and the low in winter [26]. The expression profiling of immune genes and type I inner root sheath (IRS) keratin genes in the whole skin of Aohan fine wool sheep has previously been reported by our laboratory team [27,28].

However, to our knowledge, as of yet no microarray or proteomic study at a genome-wide level has been reported on protein-coding genes which are possibly responsible for regulating hair growth of adult sheep so far. The aims of the present work are to investigate and compare the gene expression level of the body side skin and groin skin using microarray and proteomic technology, and to identify the possible genes and proteins responsible for the wool growth regulation of Aohan fine wool sheep.

## Results

### Summary of microarray analysis

A total of 1494 probes were differentially expressed comparing the body side to groin skins (S/G) in Aohan fine wool sheep, including 602 up-regulated and 892 down-regulated probes, as shown in Additional file 1: Table S1. Most probes (1110) were not assigned to unique transcripts, due to the lack of information. The number of distinct genes/transcripts (annotated) was 331, of which 112 were up-regulated and 219 were down-regulated. In S/G, 7 genes (*CYP1A1, LOC100137068, LOC443300, LOC101106865, Connexin 43, SCD and LOC101122398*) were down-regulated by more than 10-fold.

Furthermore, many gene families which regulate different aspects of hair follicle growth showed differential expression in S/G (see Additional file 2: Table S3), such as growth factors, immune cytokines, *Keratins (KRT) and Keratin-associated proteins (KAP)*, and so on.

### Selective verification for microarray data by QPCR

**Figure 1:**
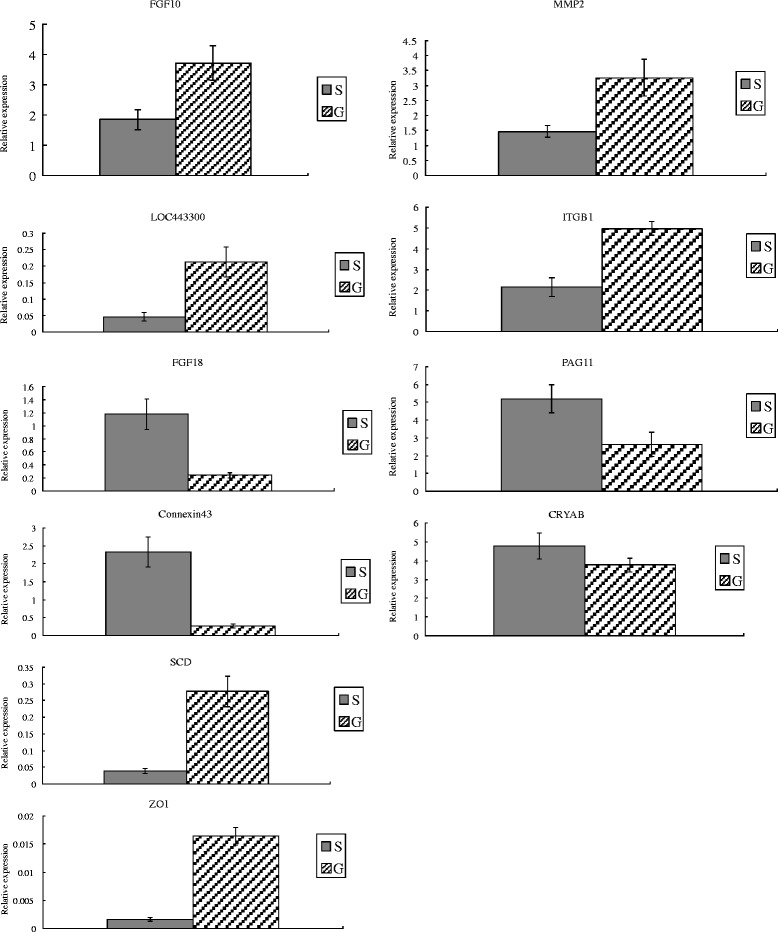
**Q-PCR validation of the microarray data.** P values (*T*-test) of the Q-PCR data are 0.036 (FGF10), 0.019 (LOC443300), 0.028 (FGF18), 0.018 (Connexin 43), 0.044 (SCD) and 0.019 (ZO1), respectively. S/G represent body side skin group/groin skin group, respectively.

In order to verify the microarray results, we selected 10 genes, namely *FGF10, LOC443300, FGF18, Connexin43, SCD*, *ZO1*, *MMP2, ITGB1, PAG11* and *CRYAB*, to comparatively analyze their expression patterns by qPCR. As shown in Figure 1, the qPCR results for the selected seven genes were consistent with the microarray results, except for *FGF10, ZO1* and *CRYAB*, thus reflecting the reliability of our microarray data.

### Hierarchical cluster analysis, biological process gene ontology (GO) analyses and putative gene networks

**Figure 2:**
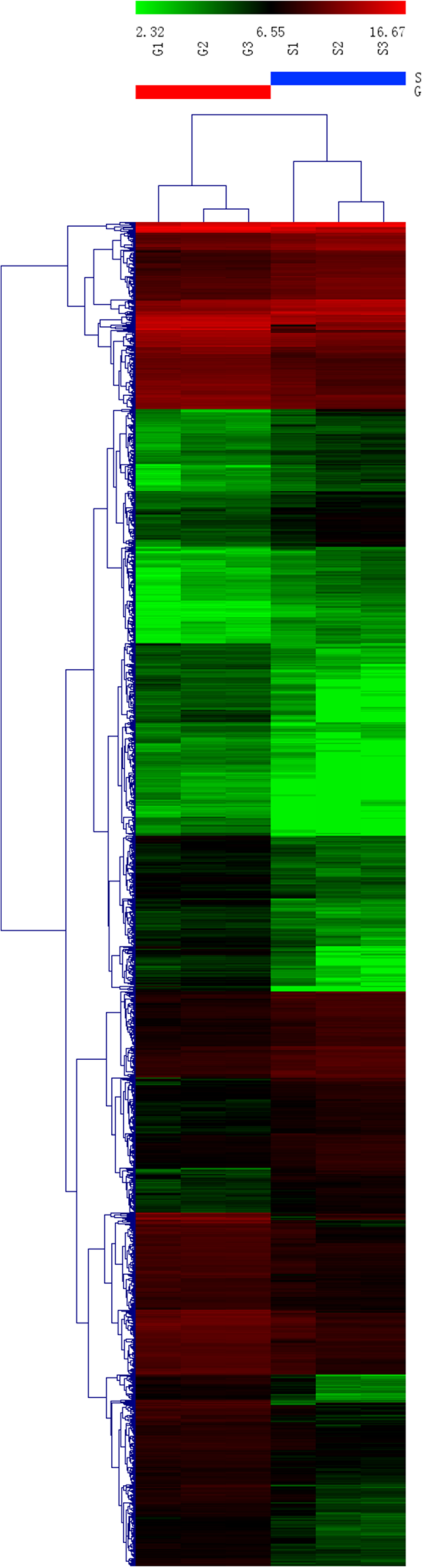
**Hierarchical cluster analysis of data between different skin areas of the Aohan fine wool sheep in anagen phase.** Each column represents one sheep, and each horizontal line refers to a gene. Color legend is on the top of the figure. Red indicates genes with a greater expression relative to the geometrical means, green indicates genes with a lower expression relative to the geometrical means. S1, S2 and S3 represent 3 repeats of body side skin group, and G1, G2, G3 represent 3 repeats of groin skin group.

To further investigate the similarity in the expression patterns of protein-coding genes between the two skin areas, we performed cluster analysis using the Cluster 3.0 tool. As shown in Figure 2, cluster analysis could make a distinction between the body side skin and the groin skin.

Based on the Database for Annotation, Visualization and Integrated Discovery (DAVID), 38 of the differentially expressed genes were classified into four categories, many of which shared the same genes, according to their functional correlation (Additional file 3: Table S2). The majority of the genes possibly related to the wool growth control could be assigned into four categories including regulation of receptor binding, multicellular organismal process, protein binding and macromolecular complex.

**Figure 3:**
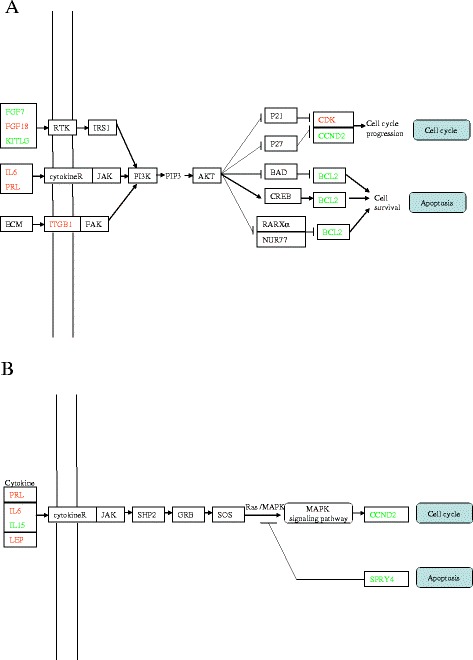
**Biological pathways having more differentially expressed genes. A**: PI3K-AKT Pathway; **B**: JAK-STAT Pathway. Gene name in red in the gene box indicates higher gene expression in S/G, green indicates lower gene expression, and black indicates no change of the gene expression.

Two singnaling pathways, PI3K-AKT pathway and JAK-STAT pathway, were identified as biological pathways having more differentially expressed genes. Figure 3 displayed the putative interactions related to the differentially expressed genes of the two pathways. These interactions were intensively involved in cell cycle and apoptosis processes.

**Figure 4:**
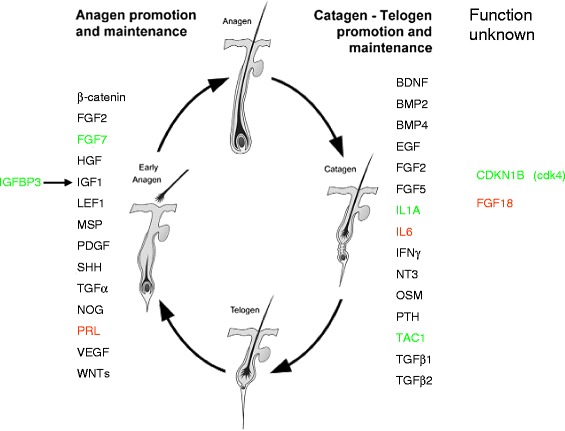
**Selected factors with possible hair growth regulatory roles** [29]**.** In the hair growth cycle, some factors play roles in anagen promotion or maintenance (left), some factors play roles in catagen–telogen promotion or maintenance (middle), and others have unkown funtions (right). The differentially expressed genes in this study was highlighted in colour (green or red). Genes in green represent that their regulation direction were in agreement with their putative function, while genes in red represent the opposite situation.

We speculate that wool growth regulation shared similar gene networks with hair. So particular differentially expressed genes, which also appeared in the conclusion of factors with known hair growth regulatory roles [29], were included in the putative gene lists of wool growth regulation (Figure 4). In the networks, 3 genes (FGF7, IGFBP3 and PRL) were contained in the category of anagen promotion and maintenance, 3 genes (IL1A, IL6 and TAC1) were contained in the category of catagen-telogen promotion and maintenance, while 2 genes (CDKN1B and FGF18) were contained in the category of function unknown.

### Quantitative comparison and identification of protein spots on 2-DE gels

**Figure 5:**
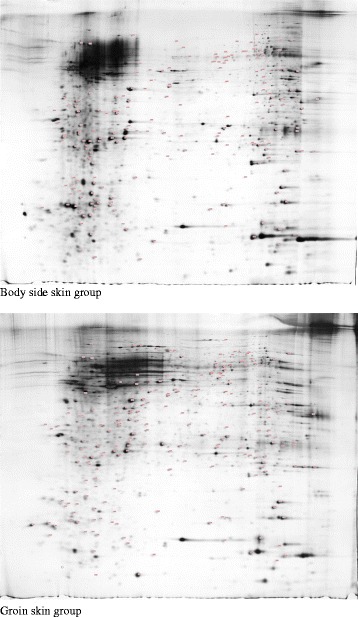
**Representative image of 2-DE silver stained polyacrylamide gel.**

To detect differential protein expression between the two skin areas, we constructed triplicate 2D maps of protein samples for each group were created. Figure 5 shows two representative 2-DE gel images of the protein expression patterns of the two groups. One hundred and eighty-seven protein spots showed significant (p < 0.05, *t*-test) differences in expression levels between the body side skin group and the control. Of the 187, 85 protein spots were more highly expressed and 102 were less highly expressed in body side skin as compared to the groin skin. Some of them could not be identified because of incomplete polypeptide fragments or low abundance (beyond the identification limit). In total, 46 protein entries were identified by MALDI-TOF/MS analyses. An overview of these proteins is presented in Additional file 4: Table S4.

## Discussion

### Methodologies

This study is a pilot to establish microarray methodologies in the wool growth field. Our results contrasted the gene expressed in the skin cells from hair-rich and hairless regions at transcriptional and translational levels. The number and fold-change of DE genes in December were much lower than that in August (5605 probes were differentially expressed in August; data not shown). That was compatible with reduced follicle activities in winter [26,30]. And most of DE genes in December were also differentially expressed in August (data not shown).

The advantage of this study is that samples could be obtained easily while the weakness of this study is the low specificity in the samples because the skin samples compose not only wool follicle, but also other parts of the skin.

### The limitations encountered during the study and how we determine the results

The small sample size is another weakness of this study. These three animals used in the study were half sibs (sharing the same father). The number of our resource population was not very large, so it was difficult to select more than three even-aged half sibs. It is unusual using only three animals, but this shortcoming could be partly made up for through using genetically related animals. Additionally, to confirm the reliability of the results from the microarray experiments, we conduct the qPCR validation for the expression levels of selected 10 genes, whose fold-changes between the two skin areas ranged from 2.15 to 19.42. Seven of these 10 genes displayed the same regulation directions compared to the microarray results, indicating our differential expression data had relatively reliable value in the field.

The technical approach (microarrays) is another limitation of the study. First, the number of the probes (15208) designed by Agilent is limited compared to the RNA-seq method [25]. Most of the probes were inadequately annotated. The full utility of the microarray data depends on the complete annotation of the whole sheep genome. Second, the specificity of the probes is relatively lower than RNA-sequencing, although using 60-mers long probes in our study. False positive and false negative phenomena exsit unavoidably in the microarray data.

### Functional implication of differentially expressed genes in the microarray study

In the present study, we investigated the molecular events possibly related to sheep hair growth control using microarray and proteomic technologies. Transcriptomic analysis identified hundreds of differentially expressed genes displaying over 2-fold difference between the two sampled skin regions of Aohan fine wool sheep at December time point, and the number of less highly expressed transcripts (892) in body side skin was greater than that of the more highly expressed ones (602). The qPCR results validated the reliability of our microarray data.

IL-1A and IL-1B inhibit hair growth in vitro as reported previously [31], but IL-1A has shown downregulation in body side skin in our study. One possibility is in vivo IL-1A controls wool growth in a time dependent or a negative feedback manner.

The skin of skin-specific *SCD1* knock-out (SKO) mice exhibited variable orthokeratotic hyperkeratosis and parakeratotic hyperkeratosis, occasional mal-aligned hair follicles, and instances of protrusion of hair shafts out of the hair follicle and into the surrounding connective tissue, with an infiltration of inflammatory cells surrounding the exposed hair shaft and keratin material [32]. In our study, *SCD* was less highly expressed at 11.91 fold change in body side skin. This maybe indicate that the *SCD* could play an important role in groin skin.

*MMP2* activity is associated with the disappearance of collagen VII during the invasion of epithelial cords of hair follicles and sweat glands in human skin [33]. *MMP2* plays a role in hair growth-associated extracellular matrix remodeling and cell migration, and may be a downstream effector through which thymosin ß4 exerts its effect on hair growth [34]. However, *MMP2* showed significant lower expression in body side skin. This may suggest sheep has a different hair growth regulatory mechanism than human.

There is a log linear relationship between the relative level of beta 1 integrins (*ITGB1*) on the cell surface and proliferative capacity in keratinocytes [35]. *ITGB1*-mediated signalling is also important in human hair growth control [36]. Skin and hair follicle integrity is crucially dependent on *ITGB1* expression in keratinocytes [37]. In our study, however,*ITGB1* expression in the body side skin was less highly expressed. The mechanism how it happens deserves further investigation.

Rowe et al. confirmed that predominantly *CYP1A1* was located to the sebaceous gland surrounding the hair shaft [38]. It is not known why *CYP1A1* expression was less highly expressed more than 10 folds in the body side skin from our data. This interesting phenomenon is worth pursuing in future studies.

As development proceeds, *GluD1* expression becomes restricted to the hippocampus, cochlear and vestibular hair cells, and spiral ganglion cells [39-41]. Hair follicles are also derived from ectoderm. So why *GLUD1* was less highly expressed in body side skin really deserves further investigation.

*Connexin 43* (*Cx43*) is a gap junction protein expressed in the follicular dermal papilla (DP) [42]. A recent mouse model research suggested an important role for *Cx43* in hair regeneration, growth, and cuticle formation [43]. However, Connexin 43 was less highly expressed in body side skin in the microarray analysis of this study, and it was also confirmed by qPCR.

Several papers indicated that prostaglandin induced hair growth [44,45], but in our results, one prostaglandin synthase (*PGFS*) were less highly expressed in body side skin. This interesting contradiction remains to be further elucidated.

We have indeed tried our best to find out differentially expressed genes associated with LCE and MOGAT as reported in a Science paper [25]. However, we could find only a few genes (DGAT2, LPL, ZO1, GJB2 and ITGB1) that have the possibility to play roles in epidermal development complex (EDC) or skin lipid metabolism. One reason for limited DE genes is that what we compared were different regions of sheep skin tissue (which might share similar principles in EDC regulation and lipid synthesis), not distinct tissues such as rumen and skin. That might also be caused by inadequate annotation of the probes or less gene coverage redundancy of the Agilent microarray.

### Functional implication of differentially expressed proteins in the proteomic study

Most of the differentially expressed proteins were not listed above in the Discussion.

Cryab protein was detected in 2DE in groin skin but not in body side skin. But the Cryab transcripts was more highly expressed in body side skin by the microarray study, though the differential expression was not validated by qPCR. This gene, downregulated in the transcriptional level during the depilation-induced hair cycle [46], was considered to play a role in regulation of apoptosis.

FGF18 protein was less highly expressed in body side skin compared to the control. But the FGF18 transcripts was more highly expressed by the microarray study. Consistent to our microarray results, FGF18 is highly expressed in hair follicles and capable of inducing anagen from telogen stage hair follicles [30].

KRT14 and KRT2.11 proteins were both less highly expressed in body side skin compared to the control. But these proteins were expected to play roles in wool follicles [47-51].

PADI3 played roles in the assembly of a globular S100A3 homotetramer,a putative Ca^2+^ modulator maturing human hair cuticle [52]. But the PADI3 protein was less highly expressed in body side skin compared to the control in our study. This interesting contradiction remains to be further elucidated.

*FGFR3* RNA was detected in precuticle cells in the periphery of the hair bulb [53,54]. But FGFR3 protein was less highly expressed in body side skin compared to the control in our study.

### Concordance between the results of transcriptomic and proteomic levels

In the results of transcriptomic level (microarray), the number of more highly expressed genes was less than that of less highly expressed ones (602 versus 892). Consistently, more highly expressed protein spots were less than more lowly expressed ones (85 versus 102).

The concordance between these two results from microarray and proteomic experiments was shown in Additional file 4: Table S4. Of the 46 identified proteins, 13 corresponding transcripts also shown differential expression. Among these 13 transcripts, the regulation trends of only 6 (in green) were consistent with their protein counterparts. The inconsistency of the two levels of other 7 DE genes (in red) might be produced by post transcriptional regulation or for unknown reasons.

### The confidence of the differentially expressed transcripts

For the Agilent microarray, the probe number of each gene is indefinite, from 1 to 5. There exists alternative splicing after gene transcription, so the inconsistency between different probes for the same gene is sometimes reasonable. Certainly the confidence is higher if all probes for the same gene were differentially expressed. For example, there are 4 probes for FGF7 and LOC443300(YWHAE) separately, of which 2 were both less highly expressed in S/G. So we speculated that the reliabilities of differential expression of FGF7 and LOC443300 were close to each other. While MMP2 differential expression was only detected in one of three probes, then its confidence might be lower than the former two genes (FGF7 and LOC443300). Nevertheless, because of the inadequate annotation of most probes, we found it difficult to calculate probe number of all the differentially expressed genes.

### Gene networks

Figure 5 listed some of the factors which possibly play roles in wool growth regulation [29]. We speculate that lower temperature and shorter day photoperiod were key causes of the downregulation of wool growth rate. So we could speculate that those 8 genes (*IL1A,FGF7,TAC1,IGFBP3,CDKN1B,IL6,FGF18 and PRL*) played roles in wool growth regulation during winter.

## Conclusions

In summary, the data presented in this study suggested that the body side skin displays a differentially expressed pattern in comparison with the groin skin at December time point. The majority of these genes possibly related to the wool growth control, and they could be assigned into the categories including regulation of receptor binding, multicellular organismal process, protein binding and macromolecular complex. Several potential gene families might participate in hair growth regulation, including fibroblast growth factors, transforming growth factor-β, insulin-like growth factor, and so on. Proteomic analysis also identified hundreds of differentially expressed proteins. This systematic analysis could lead to a better understanding of the wool growth control mechanism in Aohan fine wool sheep.

## Methods

### Animals and sample preparation

All animals were treated in accordance with the animal protocols defined by national and local animal welfare bodies, and all animal work was approved by the Shandong Province Biological Studies Animal Care and Use Committee.

Sampling methods were described previously [25,26]. One ram and two ewes of 16-month-old Aohan fine wool sheep were used in the microarray study. These animals were half sibs (sharing the same father). In December 2010, two areas of full-thickness skin were sampled from the same animal under local anaesthesia: body side skin (wool bearing) and groin skin (non-wool bearing) for microarray and proteomic experiments. The area of each sample was about 1 cm^2^. All samples were immediately put into collection tubes and stored in liquid nitrogen for RNA and protein extraction. A total of 15, 208 probes were spotted on this Agilent Sheep Gene Expression Microarray (Santa Clara, CA, USA).

### RNA extraction and microarray hybridization

TRIzol (Invitrogen) was used for total RNA extractions according to the manufacturer’s protocol. RNA quantity and quality were measured by NanoDrop ND-1000. Its OD260/OD280 ratio was confirmed to be higher than 1.8. RNA integrity was assessed by standard denaturing agarose gel electrophoresis. The RNA samples were sent to Kangchen Biotechnology Limited Company (Shanghai, China) for hybridization to the Agilent Sheep Gene Expression Microarray (Santa Clara, CA, USA). Each RNA sample was hybridized to one microarray slide. 1 μg of total RNA from each sample was amplified and transcribed into fluorescent cRNA with using the manufacturer’s Agilent’s Quick Amp Labeling protocol (version 5.7, Agilent Technologies). The labeled cRNAs were hybridized onto the Whole Genome Oligo Array (4x44K, Agilent Technologies).

### Microarrays data analysis

After hybridization and washing, the microarray slides were scanned with the Agilent Scanner G2505B. The resulting text files extracted from Agilent Feature Extraction Software (version 10.5.1.1) were imported into the Agilent GeneSpring GX software (version 11.0) for further analysis. Quantile normalization, probe annotation and subsequent data processing were performed using the GeneSpring GX v11.0 software package (Agilent Technologies). After Quantile normalization of the raw data, genes that at least 1 out of 6 samples have flags in Present (“All Targets Value”) were chosen for differentially expressed genes screening. Gene expression levels were quantified relative to the expression of GAPDH. Differentially expressed genes were identified through fold-change screening. The fold-change of 2.0 and a false discovery rate of approximately 5% were set as the threshold. All data have been deposited in NCBI’s Gene Expression Omnibus and are accessible through GEO Series accession number GSE62552 (http://www.ncbi.nlm.nih.gov/geo/query/acc.cgi?acc). Clustering analysis of all differentially expressed genes was performed using Cluster 3.0 [55,56] to analyze the similarity in the expression patterns among different skin sites. The functional annotation of differentially expressed genes was performed by the DAVID (The Database for Annotation, Visualization and Integrated Discovery) gene annotation tool (http://david.abcc.ncifcrf.gov/) [57]. The KEGG pathway analysis was done manually (http://www.genome.jp/kegg/).

Particular differentially expressed genes, which also appeared in the Figure 1 of reference No. 29 summarizing selective factors with known hair growth regulatory roles [29], were included in the putative gene networks of wool growth regulation. The networks contained genes possibly playing roles in anagen promotion and maintenance, in catagen-telogen promotion and maintenance, or with function unknown.

### QPCR

The total RNA samples prepared for microarray analysis were also used for qPCR analysis. Reverse transcriptions were performed using RevertAid First Strand cDNA Synthesis Kit (MBI Fermentas, Vilnius, Lithuania) according to the manufacturer’s protocols. The primers were designed with the Oligo 6.0 program. The primer sequences, melting temperatures and product sizes are shown in Table 1. Gene expression levels were quantified relative to the expression of GAPDH.

**Table 1:** **Primers used for Q-PCR validation**

**Gene**	**Primer sequence (5’-3’)**	**Tm (°C)** ^**a**^	**Target size (bp)**
GAPDH^b^	Forward: ATGCCTCCTGCACCACCA	60	76
	Reverse: AGTCCCTCCACGATGCCAA		
FGF10	Forward: GATCCGAGAAAGGAGCGAGG	60	554
	Reverse: TCCAGGATACTGTACGGGCA		
LOC443300	Forward: ACCAACACATCCCATTCGCT	60	140
	Reverse: CACTCAGCGTGTCCAGTTCT		
FGF18	Forward: AAGTCCGGATCAAGGGCAAG	60	98
	Reverse: CACACACTCTTTGCTGGTGC		
Connexin43	Forward: GTCGTGTCGTTGGTGTCTCT	60	291
	Reverse: CACTCAGCGTGTCCAGTTCT		
SCD	Forward: AAGAGTGGCTGAGTTTCTGGTC	60	277
	Reverse: GAAAGGAAGGTGATAGGGACAA		
Zo1	Forward: AGATAGCCCTGCAGCCAAAG	60	117
	Reverse: GGGAGGTCAAGCAGGAAGAG		
MMP2	Forward: AACGCCATCCCTGATAACCT	60	126
	Reverse: GCTTCCGAACTTCACGCTC		
ITGB1	Forward: AGCACGGATGAGGTGAACAG	60	407
	Reverse: CCAAGGCAGGTCTGACACAT		
PAG11	Forward: AGCGTCGCCTACGAATCTG	60	120
	Reverse: CTCAAACCCATATTCCGTCACA		
CRYAB	Forward: CACCCAGCTGGATTGACACT	60	147
	Reverse: CCTCATGTTTGCCATGCACC		

^a^The annealing temperature represents the optimal temperature during quantitative PCR.

^b^RNA levels of GAPDH was assayed for normalization during quantitative PCR.

### Tissue protein extraction

Lysis buffer preparation: 42% Urea, 15.2% Thiourea, 4% CHAPS, 1% DTT. Sampled tissues were homogenziated in lysis buffer (containing 1% cocktail and 2% IPG-buffer, added right before use) at the ratio of 1:7 (weight/volume). The tissues were cut into small pieces by ophthalmic scissors, and left at 4°C for 1 h, vortexed it every 15 min. Then, the tissue homogenate was centrifuge at 40,000 g for 30 min. Supernatants were collected and stored at −80°C. Protein concerntrations were determined by Bradford method.

### One-dimensional electrophoresis

0.5% IPG-buffer was added into each 150 μg protein sample (in a final volume of 400-600 μL), and was loaded in the One-dimensional electrophoresis instrument. The progamme is as follows: Step-n-hold (S1, 30 V for 6 h; S2, 60 V for 6 h); Gradient (S3, 500 V for 1 h;S4,1000 V for 1 h; S5, 3000 V for 3 h; S6, 8000 V for 3 h); Step-n-hold (S7, 8000 V for 20 h).

### 2-dimensional (2-D) SDS-PAGE preparation

Tris–HCl (PH = 8.8), Monomer storage (30% Acrylamid and 0.8% NN’-methy lenebisacry lamid), 10 × electrophoresis buffer (3.03% Tris-Base, 14.4% Glycine, 1% SDS), balanced solution (36.05% Urea, 5% Tris–HCl, 2% SDS, 34.5% Glycerine).

### The second dimensional SDS-PAGE

The electrophoresis programme is as follows:

- Transfer: Voltage 300v, Current 50 mA, Time 1 h.
- Separation: Voltage 300v, Current 200 ~ 250 mA, Time 4 ~ 5 h.
- Fixative preparation: 40% Ethanol and 10% Acetic acid.
- Electrophoresis was carried out until the blue dye front had just disappeared from the bottom of the gel.
- Fixation: take out the rubber strip and put it into Fixative for 1 h.

### Staining and visualization

Sensitizing solution preparation: 30% Ethanol, 0.314% Na_2_S_2_O_3_, 6.8%NaAc.

Sensitizing: the gels were sensitized for 30 min.

Washing: the gels were washed for three times using ddH_2_O. Ten minutes each time.

Ilver staining: Silver staining solution was prepared (1.25 AgNO3 and 200 μL Formaldehyde in 500 mL ddH_2_O). The gels were stained by the solution for 20 min.

Washing: the gels were washed for 2 min using ddH_2_O.

Visualizing solution preparation: 12.5 g Na_2_CO3 and 100 μL Formaldehyde in 500 mL ddH_2_O.

Termination solution preparation: 2 g Glycine in 50 ml ddH2O.

Visualization until the solution became muddy, then terminating for 30 min.

### Determination of relative protein expression

Gels were then scanned and analyzed using ImageMaster TM 2D platinum software (Version 5.0, GE Healthcare, San Francisco, CA, USA). The expression level was determined by the relative volume of each spot in the gel and expressed as %Vol (%Vol = [spot volume/Σvolumes of all spots resolved in the gel]). The means and standard deviations of both sample groups were calculated. Statistical significance with Student’s t-tests using ImageMaster TM 2D platinum software. P values <0.05 were considered statistically significant.

### Identification of differentially expressed proteins by mass spectrometry (MS)

Protein spots with significant differences between the two groups were excised, dehydrated in acetonitrile, and dried at room temperature. Gel pieces were denatured, alkylated, trypsin digested and analyzed by an Ultraflex II MALDI-TOF-TOF mass spectrometer (Bruker Daltonics GmbH, Bremen, Germany) under the control of FlexControl TM 2.4 software (Bruker Daltonics GmbH). Acquired peptide mass fingerprint (PMF) were processed using the software FlexAnalysis™ 3.0 (Bruker Daltonics, Bremen, Germany). The peak detection algorithm was: SNAP (Sort Neaten Assign and Place); S/N threshold: 1.5; Quality Factor Threshold: 50. The tryptic auto-digestion ion picks (trypsin [108–115] 842.5094 Da, trypsin [58–77] 2211.104 Da) were used as internal standards. The resulting peptide mass lists were used to search the Matrixscience database (http://www.matrixscience.com). The following search parameter criteria were used: mass tolerance 100 ppm, miss cleavage≦1, modification comprises carbamidomethyl and methionine oxidation. Matched peptides number between experimental PMF and theoretical PMF≧5 [58]. All MS data have been deposited in PeptideAtlas and are accessible through Dataset Identifier PASS00597 (http://www.peptideatlas.org/PASS/PASS00597).

## Availability of supporting data

All cDNA microarray data have been deposited in NCBI’s Gene Expression Omnibus and are accessible through GEO Series accession number GSE62552 (http://www.ncbi.nlm.nih.gov/geo/query/acc.cgi?acc=GSE62552).

Proteomic data have been deposited in PeptideAtlas and are accessible through Dataset Identifier PASS00597 (http://www.peptideatlas.org/PASS/PASS00597).

## Additional files

Additional file 1: Table S1. Differentially expressed genes. Additional file 2: Table S3. DE genes and their relation to different aspects of hair follicle growth. Additional file 3: Table S2. Gene Ontology analysis. Additional file 4: Table S4. Differentially expressed proteins.
