# Supplementary material for: Identification of skin-expressed genes possibly associated with wool growth regulation of Aohan fine wool sheep
Source: BMC Genet. 2014 Dec 16;15:144. doi: 10.1186/s12863-014-0144-1 (PMC4272822; doi:10.1186/s12863-014-0144-1)
Supplement: Additional file 2: Table S3. — DE genes and their relation to different aspects of hair follicle growth. [file 12863_2014_144_MOESM2_ESM.doc]

**Table S3 DE genes and their relation to different aspects of hair follicle growth (Stenn and Paus, 2001)**

| Factor Family | Gene description | probe set ID | Fold Change |
| --- | --- | --- | --- |
| ***Growth,*** ***patterning, and transcription factors*** | | | |
| *Fibroblast growth factor (FGF)* | | | |
| FGF18 | Ovis aries fibroblast growth factor 18 (FGF18) mRNA | A_70_P067996 | 3.75 |
| FGF7（KGF） | Ovis aries fibroblast growth factor 7 | A_70_P029931 | -2.26 |
| FGF7（KGF） | Ovis aries fibroblast growth factor 7 | A_70_P029932 | -3.14 |
| *Insulin-like growth factor (IGF)* | | | |
| IGFBP3 | Ovis aries insulin-like growth factor binding protein 3 (IGFBP3) | A_70_P028616 | -4.22 |
| Homeobox cluster genes | | | |
| HOXA5 | 020605ONLN139002HT ONLN Ovis aries cDNA | A_70_P017042 | -5.13 |
| HOXA5 | 020605ONLN139002HT ONLN Ovis aries cDNA | A_70_P017041 | -5.21 |
| HOXA7 | Ovis aries Hoxa-7 | A_70_P017046 | -4.38 |
| HOXA7 | Ovis aries Hoxa-7 | A_70_P017047 | -5.54 |
| *MMPs* | | | |
| MMP2 | Ovis aries matrix metallopeptidase 2 (gelatinase A, 72kDa gelatinase, 72kDa type IV collagenase) | A_70_P061386 | -2.24 |
| MMP7 | Ovis aries matrix metallopeptidase 7 (matrilysin, uterine) | A_70_P016331 | -2.09 |
| MMP7 | Ovis aries matrix metallopeptidase 7 (matrilysin, uterine) | A_70_P016332 | -3.07 |
| *Growth hormone* | | | |
| GHRHR | Ovis aries growth hormone releasing hormone receptor | A_70_P012591 | 2.35 |
| *Connexins* | | | |
| Connexin 43 | CONNEXIN 43 protein | A_70_P016371 | -19.46 |
| Connexin 43 | CONNEXIN 43 protein | A_70_P016372 | -19.43 |
| ***Cytokines*** | | | |
| *TNF* | | | |
| TNFRSF1A(Headon and Overbeek, 1999) | Ovis aries tumor necrosis factor receptor superfamily, member 1A | A_70_P036566 | -3.62 |
| *Interleukins (IL)* | | | |
| IL6 | Ovis aries interleukin 6 | A_70_P039706 | 4.06 |
| IL1A |  | A_70_P051177 | -2.84 |
| IL1A |  | A_70_P051176 | -2.44 |
| IL15 | sh3P0047E11_F.ab1 adult sheep fracture callus 10d Ovis aries cDNA, mRNA sequence | A_70_P041522 | -2.49 |
| ***Keratins（KRT） and Keratin-associated proteins（KAP）*** | | | |
| K38 | Ovis aries keratin 38 | A_70_P048717 | 4.28 |
| LOC100188976 | Sheep BIIIB3 high-sulfur keratin pseudogene mRNA | A_70_P016791 | 2.64 |
|  | Rep: Keratin, type I cytoskeletal 15 - Ovis aries (Sheep) | A_70_P058276 | -3.20 |
| SCD | Ovis aries stearoyl-CoA desaturase (delta-9-desaturase) | A_70_P039361 | -11.91 |
| ***Enzymes*** | | | |
| *Cytochrome P450* | | | |
| CYP1A1 | Ovis aries cytochrome P4501A1 | A_70_P041841 | -40.03 |
| GLUD1 | Ovis aries glutamate dehydrogenase 1 mRNA | A_70_P014771 | -4.73 |
| GLUD1 | Ovis aries glutamate dehydrogenase 1 mRNA | A_70_P014772 | -4.60 |
| *Integrins* | | | |
| ITGB1 | Ovis aries integrin, beta 1 (fibronectin receptor, beta polypeptide, antigen CD29 includes MDF2, MSK12) | A_70_P026041 | -2.81 |
| ***CD antigens*** | | | |
| CD1 | Ovis aries CD1 protein | A_70_P017086 | 3.71 |
| *Cyclin dependent* | | | |
| CDC2 | Ovis aries cyclin-dependent kinase 1 (CDK1) | A_70_P047446 | 3.71 |
| CDKN1B | 020605OCS411027046HT OCS4 Ovis aries cDNA | A_70_P024517 | -6.56 |
| CDKN1B | 020605OCS411027046HT OCS4 Ovis aries cDNA | A_70_P024516 | -3.50 |
| CCND2 | 020605ONLN034043HT ONLN Ovis aries cDNA | A_70_P005691 | 2.72 |
| CCNA2 | C0009265L09.P1KAM13F KN511 Ovis aries efferent gastric lymph from Teladorsagia infected sheep Ovis aries cDNA clone C0009265L09 5' | A_70_P034351 | 2.04 |
| *Sodium channel* | | | |
| SLC9A1 | solute carrier family 9 (sodium/hydrogen exchanger), member 1 | A_70_P031186 | 2.43 |
| *Potassium channel* | | | |
| KCNK3 | potassium channel, subfamily K, member 3 | A_70_P003551 | 2.42 |
| *Zinc ion signaling* | | | |
| ZFP36（TNF alpha inhibitor） | zinc finger protein 36, C3H type, homolog (mouse) | A_70_P030386 | -4.43 |
| *TLRs* | | | |
| TLR1 | Ovis aries toll-like receptor 1 | A_70_P049616 | 2.12 |
| *Pregnancy-associated glycoprotein* | | | |
| PAG11 | pregnancy-associated glycoprotein 11 | A_70_P050941 | 2.15 |
| *Prostaglandin(Colombe et al., 2007; Johnstone and Albert, 2002)* | | | |
| PGFS | prostaglandin F synthase | A_70_P041786 | -4.97 |
| PTGER3 | Ovis aries prostaglandin E receptor mRNA, partial cds | A_70_P041077 | 2.24 |
| *Aquaporin* | | | |
| AQP8 | Aquaporin 8 | A_70_P026742 | -4.31 |
| AQP1 | Aquaporin 1 | A_70_P051356 | -3.72 |
| AQP5 | Aquaporin 5 | A_70_P030151 | 2.30 |
| *EAAT* | | | |
| EAAT2 | Ovis aries excitatory amino acid transporter 2 | A_70_P026707 | -6.49 |
| EAAT2 | Ovis aries excitatory amino acid transporter 2 | A_70_P026706 | -6.57 |
